# Epigenetic regulation of SST_2_ expression in small intestinal neuroendocrine tumors

**DOI:** 10.3389/fendo.2023.1184436

**Published:** 2023-05-08

**Authors:** Maria J. Klomp, Julie Refardt, Peter M. van Koetsveld, Claudia Campana, Simone U. Dalm, Fadime Dogan, Marie-Louise F. van Velthuysen, Richard A. Feelders, Wouter W. de Herder, Johannes Hofland, Leo J. Hofland

**Affiliations:** ^1^ ENETS Center of Excellence, Department of Internal Medicine, Section of Endocrinology, Erasmus Medical Center (MC) Cancer Institute, Rotterdam, Netherlands; ^2^ ENETS Center of Excellence, Department of Radiology & Nuclear Medicine, Erasmus Medical Center, Rotterdam, Netherlands; ^3^ ENETS Center of Excellence, Department of Endocrinology, University Hospital Basel, Basel, Switzerland; ^4^ Endocrinology Unit, Department of Internal Medicine and Medical Specialties, School of Medical and Pharmaceutical Sciences, University of Genova, Genova, Italy; ^5^ ENETS Center of Excellence, Department of Pathology, Erasmus Medical Center, Rotterdam, Netherlands

**Keywords:** DNA methylation, histone modifications, H3K27me3, H3K9ac, epigenetic, SI-NET

## Abstract

**Background:**

Somatostatin receptor type 2 (SST_2_) expression is critical for the diagnosis and treatment of neuroendocrine tumors and is associated with improved patient survival. Recent data suggest that epigenetic changes such as DNA methylation and histone modifications play an important role in regulating SST_2_ expression and tumorigenesis of NETs. However, there are limited data on the association between epigenetic marks and SST_2_ expression in small intestinal neuroendocrine tumors (SI-NETs).

**Methods:**

Tissue samples from 16 patients diagnosed with SI-NETs and undergoing surgical resection of the primary tumor at Erasmus MC Rotterdam were analysed for SST_2_ expression levels and epigenetic marks surrounding the SST_2_ promoter region, i.e. DNA methylation and histone modifications H3K27me3 and H3K9ac. As a control, 13 normal SI-tissue samples were included.

**Results:**

The SI-NET samples had high SST_2_ protein and mRNA expression levels; a median (IQR) of 80% (70-95) SST_2_-positive cells and 8.2 times elevated SST_2_ mRNA expression level compared to normal SI-tissue (p=0.0042). In comparison to normal SI-tissue, DNA methylation levels and H3K27me3 levels were significantly lower at five out of the eight targeted CpG positions and at two out of the three examined locations within the SST_2_ gene promoter region of the SI-NET samples, respectively. No differences in the level of activating histone mark H3K9ac were observed between matched samples. While no correlation was found between histone modification marks and SST_2_ expression, SST_2_ mRNA expression levels correlated negatively with DNA methylation within the SST_2_ promoter region in both normal SI-tissue and SI-NETs (p=0.006 and p=0.04, respectively).

**Conclusion:**

SI-NETs have lower SST_2_ promoter methylation levels and lower H3K27me3 methylation levels compared to normal SI-tissue. Moreover, in contrast to the absence of a correlation with SST_2_ protein expression levels, significant negative correlations were found between SST_2_ mRNA expression level and the mean level of DNA methylation within the SST_2_ promoter region in both normal SI-tissue and SI-NET tissue. These results indicate that DNA methylation might be involved in regulating SST_2_ expression. However, the role of histone modifications in SI-NETs remains elusive.

## Introduction

Recent DNA sequencing studies have shown a very low mutation rate for well-differentiated neuroendocrine tumors (NETs) of all origins (1, 2). Accordingly, epigenetic changes are likely the principal pathological drivers in the development and progression of NETs, especially in small intestinal NETs (SI-NETs) (3, 4). Epigenetic changes affect gene expression without changing the DNA sequence and consist of DNA methylation and various histone modifications (3). DNA methylation is a process in which cytosine residues within CpG islands, which are often located in gene promoter regions, are methylated, resulting in gene silencing. Histone modifications can lead to both transcriptional repression and transcriptional activation, depending on the type of epigenetic mark and its precise location, e.g., the activating histone mark H3K9Ac and the repressive histone mark H3K27me3 (5).

Several studies have uncovered a possible prognostic role for epigenetic marks in SI-NETs. For example, promoter methylation of the *RASSF1A* and *CTNNB1* genes was associated with extensive disease and poor overall survival in SI-NETs (6–8). Another study was able to identify a panel of 21 genes with an altered DNA methylation profile resulting in changes in gene expression levels in the majority of the SI-NETs, thereby enabling to discriminate SI-NETs from other gastrointestinal tract malignancies and normal gastrointestinal tissue (2). Histone modifications also contribute to tumorigenesis, with a small study demonstrating high expression of dimethylation on H3K4 in 93% of primary intestinal neuroendocrine carcinomas (9).

In accordance with the importance of epigenetic changes in tumorigenesis of NETs, research has also been focused on epigenetic drugs to improve diagnosis and therapy of NETs. As no genetic mutations in the somatostatin receptor subtype 2 (*SST_2_*) gene have been described, it has been suggested that the epigenetic machinery is strongly involved in regulating SST_2_ expression. SST_2_ is the most important molecular marker for NETs as functional imaging with radiolabeled somatostatin analogues is crucial for tumor staging. Furthermore, sufficient SST_2_ expression is the key element for treatment with unlabeled or radiolabeled somatostatin analogues (10). Several *in vitro* and *in vivo* studies showed an increase in SST_2_ expression levels by decreasing DNA methylation and augmenting histone acetylation levels of the SST_2_ gene promoter region in human NET cell lines (11–17). Although the majority of these studies have been performed using pancreatic NET cell lines, similar effects were also observed in the SI-NET cell line GOT-1. Accordingly, one would expect correlations between epigenetic marks and SST_2_ expression levels in SI-NET tissues, i.e. inverse correlations of both DNA methylation levels and/or inhibiting histone marks with SST_2_ expression levels, and a positive correlation of SST_2_ expression with activating histone marks near the SST_2_ promoter region. However, so far, no such data have been described on SI-NETs. Therefore, the aim of this study was to investigate the role of DNA methylation as well as repressive and activating histone modifications (i.e. H3K27me3 and H3K9ac, respectively) in the regulation of SST_2_ expression of SI-NETs.

## Methods

### Samples

The selected samples consisted of fresh frozen tissue (FFT) material and formalin-fixed paraffin-embedded (FFPE) material of patients diagnosed with SI-NETs who underwent surgical resection of the primary tumor at the Erasmus MC Rotterdam, the Netherlands, and for which the diagnostic evaluation had been completed. Patients could refuse the use of their material, however, no specific consent was needed as long as patient anonymity is guaranteed.

In total, 21 SI-NET and 13 normal SI-tissues samples were collected for evaluation. Whereas FFPE material was used for SST_2_ immunohistochemistry, FFT material was used for all other analyses. Prior to analyses, FFT was cut according to standard protocol, and hematoxylin and eosin staining was performed for quality control. Based on this staining, tumor cell content was measured by counting the number of cell nuclei and, subsequently, the tissues with less than 50% tumor cell content (n=5) were excluded. Of the remaining 16 SI-NET samples, 9 had matching normal SI-tissue available.

### Immunohistochemistry

SST_2_ immunostaining was performed on 4 µm thick whole slide sections from FFPE embedded tissue blocks, on a validated and accredited automated slide stainer (Benchmark ULTRA System, VENTANA Medical Systems, Tucson, AZ, USA) according to the manufacturer’s instructions. Briefly, following deparaffinization and heat-induced antigen retrieval (pH 9.0), the tissue samples were incubated with rabbit anti-SST2A antibody (Biotrend; NB-49-015-1ML, dilution 1:25) for 32 min at 37°C, followed by Optiview detection (#760-500 & #760-700, Ventana). Counterstain was done by hematoxylin II for 12 min and a blue colouring reagent for 8 min. Stained slides were scanned with the NanoZoomer 2.0 HT (Hamamatsu Photonics, Hamamatsu City, Japan) and both the percentage of SST_2_ positive cells and the intensity per area (intensity/area) were assessed using the CellProfiler software (version 4.0.7, www.cellprofiler.org) as previously described (18).

### SST_2_ mRNA analysis

Tissues were lysed and incubated with Dynabeads oligo(dT)_25_ (Invitrogen, Breda, The Netherlands) to isolate poly-A^+^ mRNA, as described previously (17). H_2_O (23 µL) was added for elution, and 10 µL poly-A^+^ mRNA was used in the next steps. Poly-A^+^ mRNA was converted into cDNA using the commercial RevertAid First Strand cDNA synthesis kit (Thermo Scientific, Breda, Netherlands). cDNA was also prepared without the addition of RevertAid Reverse Transcriptase to exclude possible DNA contamination. Subsequently, samples were diluted by adding 180 µL H_2_O. Afterwards 5 µL sample was mixed with 7.5 µL Taqman Universal PCR mastermix (Applied Biosystems, Breda, Netherlands) supplemented with primers and probes. SST_2_ expression was determined relative to the three housekeeping genes (HKGs) *GUSB, HPRT1* and *ACTB*. Primer information can be found in **Supplemental Table 1**. For analysis, the QuantStudio 7 Flex RT-qPCR system with QuantStudio Real-Time PCR software v1.5 was used. The number of copies for SST_2_ and all HKGs was calculated by the efficiency factor to the power of ΔCt (i.e., 40 minus measured Ct). Subsequently, the relative SST_2_ expression was calculated by dividing the number of SST_2_ copies by the geometric mean of all HKGs.

### DNA isolation, bisulfite treatment and pyrosequencing

DNA was isolated from the FFT samples according to protocol of the Genome Wizard DNA isolation kit (Promega Corporation, Madison, USA). For bisulfite conversion 1000 ng DNA was used with the Zymo Research EZ DNA Zymo kit according to the manufacturer’s protocol (Zymo Research Corporation, Irvine, USA). Primer design was done with PyroMark Assay Design 2.0 (Qiagen N.V., Venlo, Netherlands). Bisulfite treated DNA was aliquoted and stored at -20°C.

Pyrosequencing of bisulfite treated DNA was performed with the primers listed in **Supplemental Table 1**. PCR products were analysed on the PyroMark Q24 (Qiagen) with PyroMark Gold Q24 reagents (Qiagen) according to manufacturer’s protocol. The eight CpG sites present in the SST_2_ promoter region were targeted, as these loci had been shown to be involved in the regulation of SST_2_ expression (19).

### Chromatin immunoprecipitation

Chromatin immunoprecipitation (CHIP) analysis was performed on 11 SI-NET samples and 13 normal SI-tissue samples, of which seven samples were matched, measuring H3K27me3 and H3K9ac enrichment at three positions of the SST_2_ promoter region, i.e. the transcription start site (TSS) and two regions upstream of this location, allocated as -2 and -1. The fold-enrichment was calculated using the following formula: *efficiency factor* ^ (CT _input adjusted_ – CT _immunoprecipitation_) x 100%, and subsequently divided by the fold-enrichment obtained with the IgG antibody. The used efficacy factors are 1.96, 1.99 and 2.00 for -2, -1 and TSS, respectively. A detailed protocol can be found in the **Supplemental Appendix**.

### Statistics

Categorical data are presented as frequencies and percentages; quantitative data are reported as mean ± standard deviation (SD) or as median and interquartile range (IQR). To test for normality, the D’Agostino and Pearson test was used. For differences between SST_2_ expression levels in SI-NET and normal SI-tissue, a paired parametric t-test was performed. For differences in epigenetic marks, a Friedman test (matched, non-parametric One-Way ANOVA) was performed with a Dunn’s multiple comparisons test. For correlation analysis, the data was log transformed to stabilize the variance, followed by Spearman correlation analysis. To test for uniform epigenetic modifications across the SST_2_ promoter region, a Spearman correlation matrix was performed on log-transformed data, using an adjusted p-value based on a Bonferroni correction. Differences were considered statistically significant at p<0.05. Statistical evaluation was performed using GraphPad Prism version 9.0.0 (GraphPad Software).

## Results

### Patient characteristics

Of the included 16 SI-NET samples, 9 (56%) came from male patients. Median age (IQR) was 61 years (54-66) at the time of tumor resection. The majority of samples were grade 1 tumors (12, 75%), while the remaining samples were low-grade 2 tumors. Nine (56%) patients had stage IV disease with lymph node metastases in 13 (81%), liver metastases in 8 (50%), bone metastases in 2 (13%) and peritoneal metastases in 4 (25%) patients. Ten (63%) patients suffered from hormonal syndrome, with 4 (25%) patients being pre-treated with somatostatin analogues of which 2 (13%) were also treated with peptide receptor radionuclide therapy using ^177^Lu-DOTATATE.

### Immunohistochemistry and mRNA analyses

**Figure 1:**
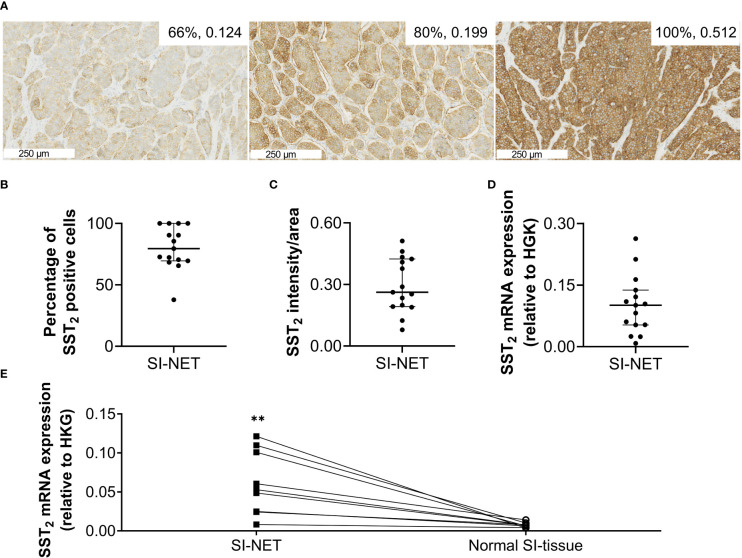
**(A)** Representative images of immunohistochemical SST_2_ staining. The numbers in the right upper corner represent the number of SST_2_ positive cells and the SST_2_ intensity/area. **(B)** The percentage of SST_2_ positive cells, **(C)** the SST_2_ intensity/area and **(D)** SST_2_ mRNA expression levels measured in small intestinal neuroendocrine tumor tissue, and **(E)** SST_2_ mRNA expression levels in small intestinal neuroendocrine tumor samples compared to paired normal small intestinal tissue. Data in **(B–D)** are presented as median with interquartile ranges. **p<0.01 SI-NET, small intestinal neuroendocrine tumor; normal SI-tissue, normal small intestinal tissue; SST_2_, somatostatin receptor subtype 2; HKG, housekeeping genes.

Overall, the SI-NET samples showed high SST_2_ expression, with a median (IQR) percentage of positive cells of 80% (70-100) and an intensity/area of 0.262 (0.192-0.424) based on SST_2_ IHC, and a SST_2_/HKG ratio of 0.10 (0.05-0.14) based on the RT-qPCR analysis, **Figures 1A–D**. Results for one sample had to be excluded from SST_2_ IHC quantification due to insufficient eosinophilic counter-staining, hampering automated analysis. Analysis of the nine matched samples showed that SST_2_ mRNA expression levels of the SI-NET tissues were on average 8.2 times higher compared to that of normal SI-tissue with a median (IQR) SST_2_/HKG ratio of 0.05 (0.02-0.10) and 0.007 (0.005-0.009), p=0.0042, for SI-NETs and normal SI-tissue, respectively, **Figure 1E**. No underlying factor such as gender, grade or stage for the wide range in expression could be identified. Also, no significant differences in SST_2_ mRNA or protein expression levels between treatment-naïve versus pretreated patients were observed (data not shown).

### Epigenetic profiles of SI-NET samples

**Figure 2:**
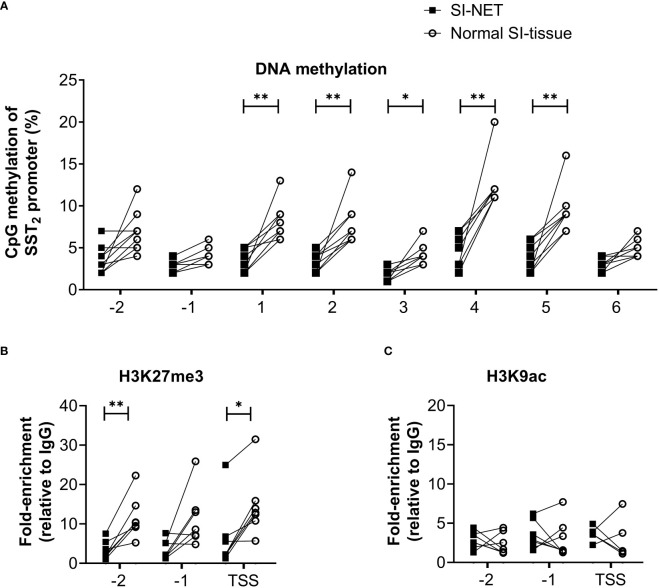
**(A)** Percentage of DNA methylation levels at different CpG positions of the SST_2_ gene promoter of small intestinal neuroendocrine tumor samples compared to matching normal small intestinal tissue. **(B, C)** Enrichment of H3K27me3 and H3K9ac on three locations in the SST_2_ promoter region (i.e. -2, -1 and TSS) in small intestinal neuroendocrine tumor samples compared to the matching normal small intestinal tissue. Data is presented as fold enrichment relative to IgG controls and log-transformed. *p<0.05, **p<0.01. SI-NET, small intestinal neuroendocrine tumor; normal SI-tissue, normal small intestinal tissue; SST_2_, somatostatin receptor subtype 2.

Using the matched tissue samples, it was demonstrated that the epigenetic profiles of SI-NET tissues differ compared to normal SI tissues. In general, DNA methylation levels of the SST_2_ gene promoter of the SI-NET samples were relatively low and significantly lower at five out of the eight targeted CpG positions compared to what was observed in the normal SI-tissue, **Figure 2A**. For SI-NET samples, we observed a uniform DNA methylation across the SST_2_ promoter region, with each location, except position –1, showing a significant positive correlation with at least three other locations (**Supplemental Table 2**). Interestingly, position -1 showed a significant positive correlation with four positions in normal SI-tissue, whereas location 6 was not characterized by any significant correlation (**Supplemental Table 3**).

In addition to DNA methylation of the SST_2_ promoter region, differences were also found in histone methylation profiles. The enrichment of repressing epigenetic mark H3K27me3 was significantly lower in two out of the three locations in SI-NET tissue compared to the matched normal SI-tissue, **Figure 2B**. No differences in the activating histone mark H3K9ac position were observed between matched samples, **Figure 2C**. Similar to the pattern observed for the DNA methylation profile, a uniform epigenetic profile was also demonstrated for the histone marks, i.e. a significant positive correlation between -2, -1 and TSS for both histone methylation and acetylation, **Supplemental Tables 4, 5**.

### Epigenetic profiles and SST_2_ expression

**Figure 3:**
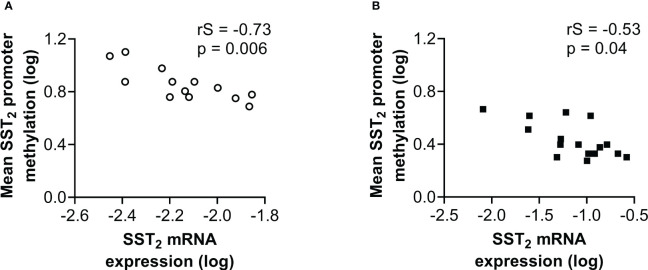
Correlation of the mean level of DNA methylation at CpG positions in the SST_2_ promoter region with SST_2_ mRNA expression levels in **(A)** normal small intestinal tissue and **(B)** small intestinal neuroendocrine tumor samples. Data are log-transformed. rS, Spearman r; SST_2_, somatostatin receptor subtype 2.

To further evaluate the role of the epigenetic marks in regulating SST_2_ expression, the epigenetic modifications were correlated with the percentage of SST_2_ positive cells, the SST_2_ intensity/area and SST_2_ mRNA expression levels. SST_2_ mRNA expression levels correlated negatively with the mean level of DNA methylation of the SST_2_ promoter in the normal SI-tissue samples (p=0.006, **Figure 3A**), reaching statistical significance (adjusted p-value threshold of 0.006) for the individual CpG positions 1, 2 and 4 (rS = -0.79, -0.81 and -0.74; p=0.002, 0.001 and 0.005, respectively). For the SI-NET samples, a statistically significant negative correlation was also found for SST_2_ mRNA expression levels and the mean level of DNA methylation of the SST_2_ promoter (p=0.04), **Figure 3B**. However, using the adjusted p-value threshold of 0.006, no individual location showed a significant correlation, but a trend towards negative correlations was observed for location 1, 3, 4 and 5 (rS = -0.59, -0.58, -0.52 and –0.61; p=0.019, 0.019, 0.040 and 0.013, respectively). No statistically significant correlations between the mean level of DNA methylation and the number of SST_2_ positive cells (p=0.41) nor SST_2_ intensity/area (p=0.21) were demonstrated in SI-NET tissues (**Supplemental Figure 1**).

**Figure 4:**
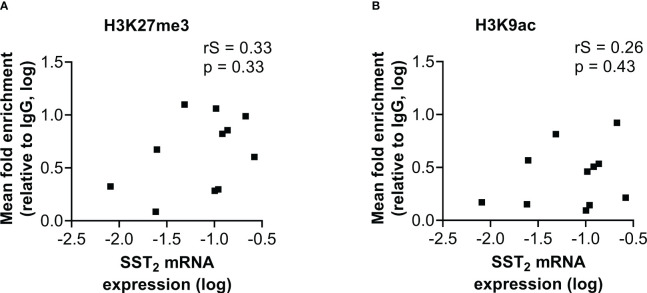
Correlation of SST_2_ mRNA expression levels with the fold enrichment of **(A)** H3K27me3 and **(B)** H3K9ac calculated as the mean enrichment on three locations within the SST_2_ promoter (i.e. -2, -1 and TSS) in the small intestinal neuroendocrine tumor samples. All data are presented as fold enrichment relative to IgG and data are log-transformed. rS, Spearman r; SST_2_, somatostatin receptor subtype 2.

A similar correlation analysis was performed with the mean level of histone mark enrichment on the three examined locations within the SST_2_ promoter region. In contrast to the correlation found between the level of DNA methylation and SST_2_ mRNA expression in both normal SI-tissue and SI-NETs, no correlations were found in SI-NET samples between histone mark enrichment and SST_2_ mRNA expression levels (p=0.33 and p=0.43 for H3K27me3 and H3K9ac, respectively), **Figure 4**, nor with the percentage of SST_2_ positive cells (p=0.54 and p=0.89 for H3K27me3 and H3K9ac, respectively), or the SST_2_ intensity/area (p=0.19 and p=0.71 for H3K27me3 and H3K9ac, respectively, **Supplemental Figure 2**). Whereas correlations using the mean level of enrichment were lacking, correlations were also not found focusing for each individual location.

## Discussion

The aim of the current study was to investigate the association between DNA methylation, histone modifications and SST_2_ expression in SI-NET tissues. We showed that the SI-NET tissues had lower DNA and histone methylation levels compared to normal SI-tissue. Moreover, significant negative correlations were found between SST_2_ mRNA expression level and DNA methylation levels within the SST_2_ promoter region for both normal SI-tissue and SI-NETs.

Our results confirm that DNA methylation may play a role in SI-NET tumorigenesis. DNA methylation levels are significantly lower in the SI-NET samples compared to the adjacent normal SI-tissue, suggesting tumor induced changes in the epigenetic profile of the SST_2_ promoter region. In addition, we were able to show a clear negative correlation between the mean level of DNA methylation within the SST_2_ promoter and SST_2_ mRNA expression level in SI-NETs. Although significance was not reached after correcting for multiple testing, locations 1, 3, 4 and 5 seemed to be mostly involved in regulating SST_2_ expression. It cannot be excluded that a higher sample size might have led to significant results in one or multiple of these individual locations. Moreover, the heterogeneous character of SI-NET tissues could have complicated the analysis (20). The observed negative correlation is in line with previous research using NET cell lines showing compelling results demonstrating upregulation of SST_2_ expression following epigenetic treatment, and more specifically, DNA methyltransferase inhibitors (12, 21). Moreover, a significant inverse correlation was found between DNA methylation – measured within an CpG island containing an upstream TSS for SST_2_ – and SST_2_ mRNA expression levels in a panel of 11 cell lines (19). We did not only demonstrate a correlation between DNA methylation and SST_2_ mRNA in SI-NETs, a correlation was also found in normal SI-tissue. Surprisingly, location -1 was not correlated with any other positions in SI-NETs, whereas this was position 6 in normal SI-tissue. It is therefore possible that the epigenetic machinery responsible for DNA methylation is activated differently in normal SI-tissue and SI-NET tissue. Nevertheless, it should be considered that for a true comparison enterochromaffin cells should have been analysed instead of the normal SI-tissue.

While a correlation between DNA methylation and SST_2_ mRNA expression was found in the SI-NET tissues, this correlation was not found between DNA methylation levels and the percentage of SST_2_ positive cells, nor with the intensity/area. This might be due to the analyses performed; whereas mRNA and DNA methylation levels were determined based on the entire tumoral tissue including other cell types (e.g. fibrotic cells, endothelial cells), quantification of the SST_2_ IHC was purely based on the analysis of tumor cells. Also, while mRNA and DNA methylation were both studied from FFT, protein expression was quantified on FFPE samples, possibly introducing a sample bias. It would therefore have been of interest to perform western blot analysis on FFT material as well. Unfortunately, this analysis could not be performed due to the scarcity of tissue, and no statement can be made about possible correlations.

In contrast to the correlations found between SST_2_ mRNA and DNA methylation, no correlations were found between two widely studied histone modifications, i.e. activating (H3K9Ac) and repressive (H3K27me3) histone marks, and SST_2_ expression levels. Possibly other epigenetic histone modifications are involved that can alter SST_2_ gene expression, e.g. histone methylation at H3K9me2/3 (repressing), or at H3K4me1/2/3 and H3K36me3 (activating). Moreover, several lysine residues can be acetylated resulting in activation of gene transcription (22). Accordingly, the use of antibodies for panacetylation on either histone 3 or histone 4 might be of interest, thereby evaluating histone modifications in a broader view.

Research is currently focusing on upregulating SST_2_ in NETs to improve diagnosis and treatment, but the available clinical data is ambiguous. Based on our findings, we would expect epigenetic drugs targeting the DNA methylation profile to be more effective in upregulating SST_2_ than drugs targeting the histone modifications. However, one trial involving nine patients with NETs from different origin and low baseline SST_2_ expression showed no SST_2_ upregulation upon epigenetic treatment with DNA methyltransferase inhibitor hydralazine combined with histone deacetylase (HDAC) inhibitor valproic acid (23). Meanwhile, another small clinical trial involving five well-differentiated SI-NET patients with sufficient SST_2_ expression showed a minor but significant increase in radiolabelled somatostatin analogue uptake after treatment with the HDAC inhibitor vorinostat (24). As discussed above, different histone marks could play a role in SST_2_ upregulation, thereby enabling SST_2_ upregulation in response to vorinostat. The opposing outcomes of these two clinical studies could also be due to differences in intratumoral drug levels or differences in tumor biology between NETs with low and high SST_2_ expression (25).

Our current study only focused on SI-NETs, and it is therefore unknown if our findings would have been similar in NETs of other origins. In line with our results, a correlation was found between the level of DNA methylation in the SST_2_ promoter and SST_2_ expression levels in pancreatic NETs (26). In contrast, the direct role of histone marks in regulation SST_2_ in pancreatic NETs remains unclear. *In vitro* experiments using pancreatic NET cell lines, e.g. BON-1 and QGP-1, showed convincing effects of HDAC inhibitors on SST_2_ expression (17, 21, 23). Moreover, elevated HDAC expression levels have been described in pancreatic NET tissues (27), together suggesting a possible role of histone acetylation in regulating SST_2_ expression in pancreatic NETs. However, despite these data, evidence for a direct association is lacking.

In conclusion, our study showed that well-differentiated SI-NETs have lower DNA and histone methylation levels on the SST_2_ promoter region compared to normal SI-tissue. A statistically significant correlation between SST_2_ mRNA expression and DNA methylation within the SST_2_ promoter region was observed in both normal SI-tissue and SI-NETs. Thus, while epigenetic factors seem to play an important role in SI-NET tumorigenesis, it is mainly DNA methylation that seems to be involved in regulating SST_2_. However, the role of histone modifications in regulating SST_2_ expression remains to be further elucidated.

## Data availability statement

The data supporting the conclusion of this article will be made available by the authors upon reasonable request.

## Ethics statement

Ethical review and approval was not required for the study on human participants in accordance with the local legislation and institutional requirements. Written informed consent for participation was not required for this study in accordance with the national legislation and the institutional requirements.

## Author contributions

Study conceptualization: LH, JH, MK and JR. MK, JR, PK, CC and FD performed the experiments, analysed and interpreted the data. MV, SD, RF, WH, JH and LH advised on the analyses and interpretation of the data. MK and JR wrote the initial draft of this article. All authors revised the manuscript. All authors contributed to the article and approved the submitted version.
