# Supplementary material for: Epigenetic regulation of SST2 expression in small intestinal neuroendocrine tumors
Source: Front Endocrinol (Lausanne). 2023 May 8;14:1184436. doi: 10.3389/fendo.2023.1184436 (PMC10200989; doi:10.3389/fendo.2023.1184436)
Supplement: Supplementary file 1 [file DataSheet_1.pdf]

frontiers

Supplementary Material

Epigenetic regulation of SST_2 expression in small intestinal neuroendocrine tumors

†Maria J. Klomp[1,2], †Julie Refardt[1,3*], Peter M. van Koetsveld[1], Claudia Campana[1,4], Simone U. Dalm[2], Fadime Dogan[1], Marie-Louise F. van Velthuysen[5], Richard A. Feelders[1], Wouter W. de Herder[1], Johannes Hofland[1], Leo J. Hofland[1]

[1]ENETS Center of Excellence, Department of Internal Medicine, Section of Endocrinology, Erasmus MC Cancer Institute, Rotterdam, The Netherlands; [2]ENETS Center of Excellence, Department of Radiology & Nuclear Medicine, Erasmus Medical Center, Rotterdam, The Netherlands; [3]ENETS Center of Excellence, Department of Endocrinology, University Hospital Basel, Basel, Switzerland; [4]Endocrinology Unit, Department of Internal Medicine and Medical Specialties, School of Medical and Pharmaceutical Sciences, University of Genova, Genova, Italy; [5]ENETS Center of Excellence, Department of Pathology, Erasmus Medical Center, Rotterdam, The Netherlands.

†These authors contributed equally to this work and share first authorship

*** Correspondence:**
Dr. Julie Refardt
ENETS Center of Excellence
Department of Endocrinology, University Hospital Basel
Petersgraben 4, 4031 Basel, Switzerland
Julie.refardt@usb.ch

1 Supplementary Data

Chromatin immunoprecipitation

Snap frozen tissue was crushed and washed with 950 µL PBS supplemented with 1 mM phenylmethylsulfonyl fluoride (PMSF, Thermo Scientific), followed by incubation with formaldehyde for fixation (final concentration of 1%, 10 minutes, room temperature (RT)). To quench the reaction, glycine was added (final concentration of 0.125 M) and the sample was incubated (5 minutes, RT). The sample was washed twice with 950 µL PBS supplemented with 1 mM PMSF (2000 RPM, 5 minutes, 4ºC), and the pellet was then resuspended in 500 µL lysis buffer (1 % sodium dodecyl sulphate (SDS), 50 mM tris hydrochloride (Tris-HCl, pH 8.1) and 10 mM ethylenediaminetetraacetic acid (EDTA, pH 8.0) supplemented with 1 mM PMSF). After incubation on ice (10 minutes), the sample was sonified on ice for 5 - 11 times for 20 seconds at 600 microns. The sample was centrifuged (13000 RPM, 10 minutes, 4ºC) and the supernatant was used for further analysis. To include a sample for further analysis, the right DNA concentration and fragment size was confirmed. To do so, 50 µL of the supernatant was incubated (30 minutes 37ºC) with 100 µL H_2O, 6 µL 5 M NaCl and 1 µL RNase A (20 mg/mL). Then 2 µL proteinase K (20 mg/mL) was added and the sample was incubated again (4 hours, 65ºC). The DNA was purified with the QIAquick PCR purification kit (Qiagen) according to manufacturer's protocol and subsequently heated (10 minutes, 65ºC). The quality of the sample was then confirmed with the NanoDrop 8000 spectrophotometer (Thermo Scientific) and the 4200 TapeStation system (Agilent) (Supplemental Figure 3). For this, 1 µL of 50 ng/µL DNA was loaded into the Genomic DNA ScreenTape (Agilent).

Per antibody of interest, 5 µg chromatin was diluted in CHIP dilution buffer (1.1% Triton X-100, 0.01% SDS, 167 mM NaCl, 16.7 mM Tris-HCl (pH 8.1) and 1.2 mM EDTA supplemented with 1x SIGMAFAST protease inhibitor (Sigma)) to a total volume of 500 µL. Then rabbit anti-mouse IgG antibody (ab46540, Abcam) was added and the mixture was incubated (1 hour, 4ºC) to preclear the chromatin, followed by the incubation (2 hours, 4ºC) with Dynabeads Protein-G (Invitrogen). Of the precleared chromatin sample, an input sample (10%) was collected. Then, 2.5 µg antibody (H3K27me3 (ab6002, Abcam), H3K9ac (ab4441, Abcam) or IgG (ab46540, Abcam)) was added to each 5 µg chromatin sample, followed by an overnight incubation step (4ºC).

Dynabeads Protein-G was added followed by an incubation step (2 hours, 4ºC). The sample was washed: (1) three times with 20 mM Tris-HCl (pH 8.0), 2 mM EDTA, 1 % Triton X-100 and 150 mM NaCl, (2) once with 20 mM Tris-HCl (pH 8.0), 2 mM EDTA, 1 % Triton X-100, 0.1% SDS and 500 mM NaCl, (3) once with 10 mM Tris-HCl (pH 8.0), 1 mM EDTA, 0.25 M lithium chloride (LiCl), 0.5% IGEPAL and 0.5% sodium deoxycholate and (4) once with 10 mM Tris-HCl (pH 8.0) and 1 mM EDTA. Then, 150 µL elution buffer (25 mM Tris-HCl (pH 7.5) + 10 mM EDTA + 0.5% SDS) was added and the chromatin was eluted by incubation (30 minutes, 65ºC, 1200rpm). To both the input sample and the eluted chromatin, 6 µL 5 M NaCl and 2 µL proteinase K (20 mg/ml) were added and the samples were incubated (4 hours, 65ºC). The resulting DNA was purified with the QIAquick PCR purification kit (Qiagen) and the enrichment was measured by RT-qPCR. For this, 2 µL DNA was mixed with 10.5 µL primer mix consisting of 3.5 µL H_2O, 0.375 µL 10 µM reverse primer, 0.375 µL 10 µM forward primer and 6.250 µL 2x SensiFAST SYBR Lo-ROX mix (Meridian Bioscience). Primer information can be found in *Supplemental Table 1*. To obtain the CT-values, a threshold of 0.030 was used for all three primer sets (i.e. -2, -1 and TSS).

2 Supplementary Tables and Figures

Supplemental Table 1.

Table S1. (A) Primer sequences for RT-qPCR. Primers for housekeeping genes (Thermo Fisher Scientific) were diluted twenty times. For SST_2, final concentrations were 0.5 pM for both the forward and reverse primer, and 0.1 pM for the SST_2 probe (Sigma). (B) PCR and sequencing primer sequences for pyrosequencing. (C) Primer sequences for CHIP analysis (11).

	**Primer information**	**Efficiency Factor**
**(A) RT-qPCR**		
**GUSB**	Hs00939627_m1	1.95
**HPRT1**	Hs02800695_m1	1.97
**B-Actin**	Hs01060665_g1	1.96
**SST_2**	Forward:<br>5’-TCGGCCAAGTGGAGGAGAC-3’<br><br>Reverse:<br>5’-AGAGACTCCCCACACAGCCA-3’<br><br>Probe:<br>5’-FAM-CCGGACGGCCAAGATGATCACC-TAMRA-3’	1.91
**(B) Pyrosequencing**		
**PCR**	Forward:<br>5’-[Btn]GGGTTGGTTGGGTTAGTTTT -3’<br><br>Reverse:<br>5’-ATTCCTAACTCCTCCACCCTCTT-3’	
**Sequencing**	Reverse strand:<br>5’-ACCTCAAACTAAAACTCTA-3’	

(C) CHIP analysis		
**-2**	Forward:<br>5’-TGCTGACTGACGTGGCTACA-3’<br><br>Reverse:<br>5’-CGCACCTGGAGTCCAAGATT-3’	1.96
**-1**	Forward:<br>5’-GTCCTTGCCATGAGTCTTGA-3’<br><br>Reverse:<br>5’-CAGGCAGAGCTTACAGACAG-3’	1.99
**TSS**	Forward:<br>5’-AGCGAAGCCGCTGTGACGTA-3’<br><br>Reverse:<br>5’-TCTGGGCGCTGGTGGTCTTG-3’	2.00

Supplemental Table 2.

Table S2. Spearman R values of the correlation analyses between the eight examined CpG positions of the SST_2 promoter in small intestinal neuroendocrine tumors samples, demonstrating a uniform DNA methylation profile, except for location -1 which did not correlate with any other location. To correct for multiple testing, results were considered statistically significant at $p<0.002$ and are shown in bold.

	**-2**	**-1**	**1**	**2**	**3**	**4**	**5**	**6**
**-2**		0.06	**0.79**	0.72	**0.75**	**0.74**	**0.75**	0.72
**-1**			0.29	-0.18	0.27	0.08	0.08	-0.30
**1**				**0.75**	**0.92**	**0.82**	**0.88**	0.68
**2**					0.67	**0.79**	**0.88**	**0.82**
**3**						**0.83**	**0.85**	0.64
**4**							**0.90**	**0.81**
**5**								**0.83**
**6**								

Supplemental Table 3.

Table S3. Spearman R values of the correlation analyses between the eight examined CpG positions of the SST_2 promoter in normal small intestinal tissue samples, demonstrating a uniform DNA methylation profile, except for location 6 which did not correlate with any other location. To correct for multiple testing, results were considered statistically significant at p<0.002 and are shown in bold.

	**-2**	**-1**	**1**	**2**	**3**	**4**	**5**	**6**
**-2**		0.79	**0.80**	**0.83**	0.74	**0.89**	**0.80**	0.74
**-1**			**0.90**	0.73	**0.92**	**0.87**	**0.82**	0.69
**1**				**0.86.**	**0.96**	**0.95**	**0.88**	0.69
**2**					**0.80**	**0.89**	**0.91**	0.57
**3**						**0.90**	**0.81**	0.63
**4**							**0.90**	0.68
**5**								0.75
**6**								

Supplemental Table 4.

Table S4. Spearman R values of the correlation analyses between the three examined locations within the SST_2 promoter region (i.e. TSS, -2 and -1) in small intestinal neuroendocrine tumor samples, demonstrating a uniform H3K27me3 profile. To correct for multiple testing, results were considered statistically significant at $p<0.017$ and are shown in bold.

	**TSS**	**-2**	**-1**
**TSS**		**0.96**	**0.87**
**-2**			**0.95**
**-1**			

Supplemental Table 5

Table S5. Spearman R values of the correlation analyses between the three examined locations within the SST_2 promoter region (i.e. TSS, -2 and -1) in small intestinal neuroendocrine tumor samples, demonstrating a uniform H3K9ac profile. To correct for multiple testing, results were considered statistically significant at p<0.017 and are shown in bold.

	**TSS**	**-2**	**-1**
**TSS**		**0.89**	**0.89**
**-2**			**0.92**
**-1**			

Supplemental Figure 1.

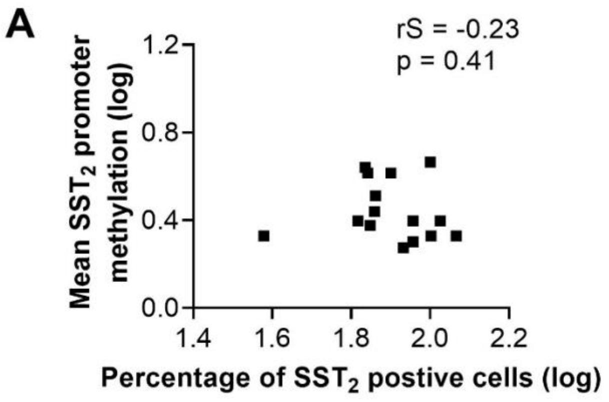

Figure S1 Correlation of the mean level of DNA methylation at CpG positions in the SST_2 promoter region with (A) the percentage of SST_2 positive cells and (B) the SST_2 intensity/area in small intestinal neuroendocrine tumor samples. Data are log-transformed.

rS = Spearman r, SST_2 = somatostatin receptor subtype 2

Supplemental Figure 2.

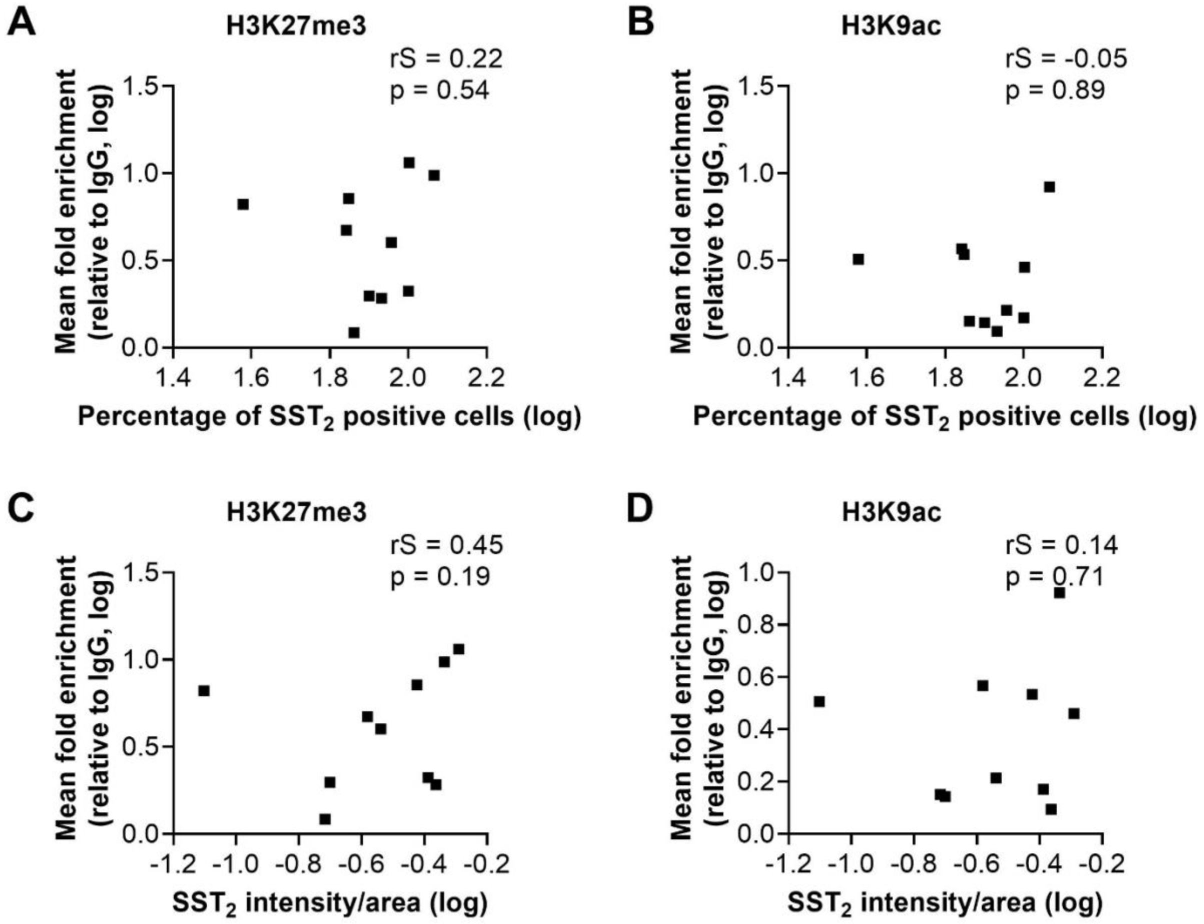

Figure S2 Correlation of (A, B) the percentage of SST_2 positive cells and (C, D) the SST_2 intensity/area with the fold enrichment of (A, C) H3K27me3 and (B, D) H3K9ac calculated as the mean enrichment on three locations within the SST_2 promoter (i.e. -2, -1 and TSS) in the small intestinal neuroendocrine tumor samples. All ChIP data are presented as fold enrichment relative to IgG and data are log-transformed.

rS = Spearman r, SST_2 = somatostatin receptor subtype 2

Supplemental Figure 3.

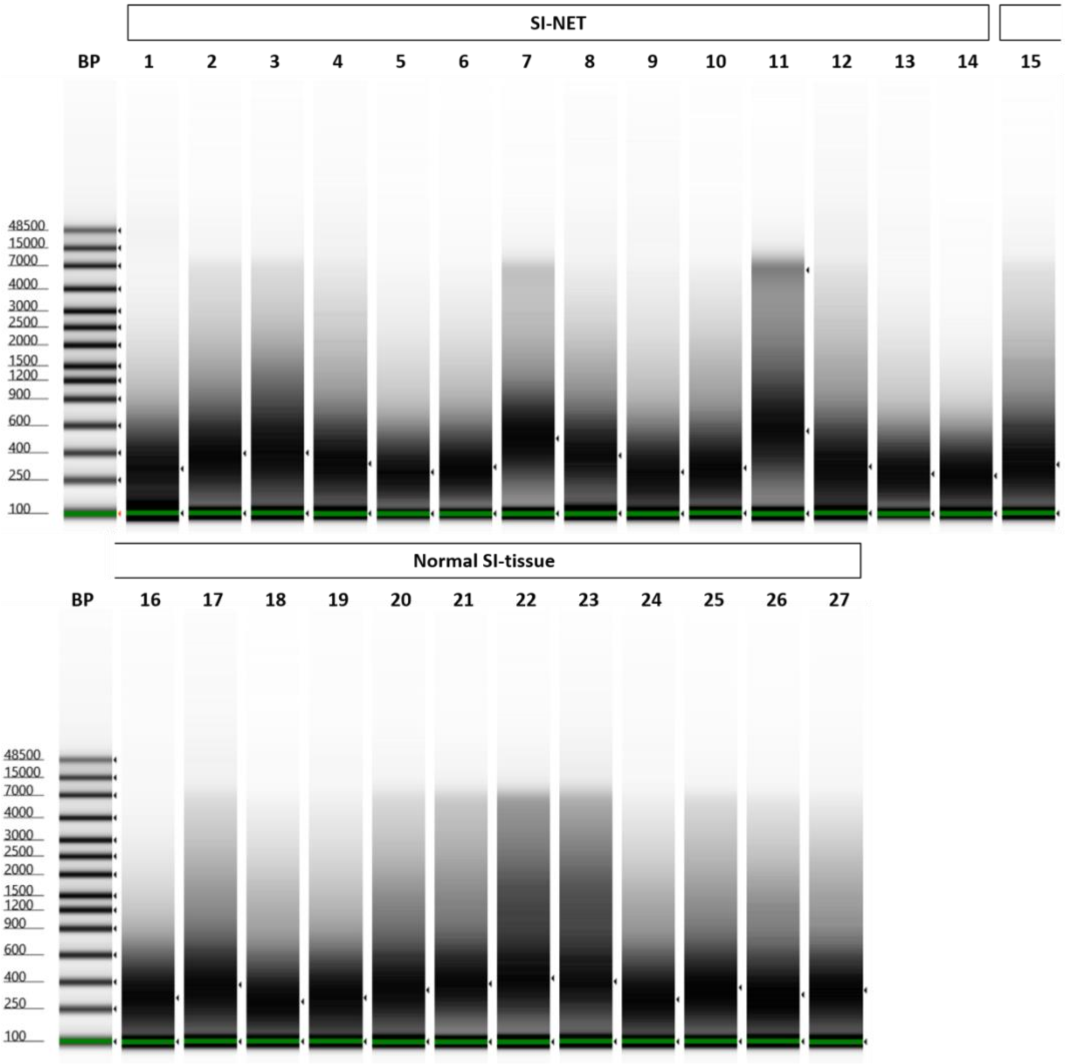

Figure S3 DNA fragment size of small intestinal neuroendocrine tumor samples and normal small intestinal tissue, demonstrating that the majority of the DNA fragments has the desired fragment size between 200 and 1000 base pairs. SI-NET = small intestinal neuroendocrine tumor, Normal SI-tissue = normal small intestinal tissue, BP = base pairs.
